# Low-intensity Extracorporeal Shockwave Therapy for the Management of Postprostatectomy Erectile Dysfunction: A Systematic Review of the Literature

**DOI:** 10.1016/j.euros.2022.07.003

**Published:** 2022-07-30

**Authors:** Maria Chiara Sighinolfi, Ahmed Eissa, Carlo Bellorofonte, Alessandro Mofferdin, Mosaab Eldeeb, Simone Assumma, Enrico Panio, Tommaso Calcagnile, Daniele Stroppa, Giorgio Bozzini, Giorgia Gaia, Stefano Terzoni, Mattia Sangalli, Salvatore Micali, Bernardo Rocco

**Affiliations:** a Urology Department, ASST Santi Paolo e Carlo, University of Milan, Milan, Italy; b Urology Department, Faculty of Medicine, Tanta University, Tanta, Egypt; c Urology Unit, Columbus Clinic Center, Milan, Italy; d Urology Department, University of Modena & Reggio Emilia, Modena, Italy; e Urology Department, ASST Lariana, Como, Italy; f Gynecology Department, ASST Santi Paolo e Carlo, Milan, Italy

**Keywords:** Low-intensity extracorporeal shockwave therapy, Erectile dysfunction, Radical prostatectomy

## Abstract

**Context:**

Erectile dysfunction (ED) following radical prostatectomy is a concern for patients and their partners. Low-intensity extracorporeal shockwave therapy (LI-ESWT) can potentially enhance tissue repair and regeneration. The aim of the current study was to systematically review the literature to assess the role of LI-ESWT in the management of patients with postprostatectomy ED.

**Evidence acquisition:**

Two authors independently performed a systematic search of the PubMed and Web of Science databases to identify all relevant articles. Non-English reports, case reports, reviews, letters, and editorials were excluded. Risk of bias was assessed according to the GRADE guidelines.

**Evidence synthesis:**

Nine articles met the inclusion criteria and were included in the qualitative analysis. All the studies included were published between 2015 and 2022 and the majority of them compared phosphodiesterase type 5 inhibitors (PDE5Is) alone versus a combination of LI-ESWT and PDE5Is. Only three studies were randomized controlled trials (RCTs). In general, there is no standardized protocol for LI-ESWT for postprostatectomy ED. In comparisons of LI-ESWT + PDE5Is versus PDE5Is alone, some authors found a statistically significant improvement in erectile function with LI-ESWT + PDE5Is. The starting time for LI-ESWT differed among the studies, ranging from 3 d to 6 mo after surgery. The main limitations of the review are the scarcity of studies, small sample sizes, high risk of bias, and high heterogeneity among studies.

**Conclusions:**

There is currently limited evidence on the use of LI-ESWT either alone or in combination with PDE5Is in penile rehabilitation protocols after prostatectomy. However, small clinical trials with short follow-up show that LI-ESWT could potentially play a role in the management of postprostatectomy ED in the future. Further RCTs with larger sample sizes are needed.

**Patient summary:**

Despite limited reports in the literature, low-intensity shockwave therapy after removal of the prostate is a promising noninvasive treatment for dealing with erectile dysfunction after surgery.

## Take Home Message

This is the first systematic review of low-intensity extracorporeal shockwave therapy for neurogenic erectile dysfunction following radical prostatectomy. Despite growing interest in the use of this technology, data in the literature on this topic are still scarce.

## 1 Introduction

A survey-based study showed that erectile dysfunction (ED) is the most distressing problem after radical prostatectomy (RP) over the long term, the impact of which on patients’ quality of life is greatly underestimated by surgeons [1]. Considering the important role of the neurovascular bundle (NVB) located at the posterolateral aspect of the prostate, a nerve-sparing approach to RP was proposed in the early 1980s to enhance postoperative erectile function [2], but ED has persisted as a life-distressing sequala of RP for patients and their partners [1]. This is because nerve injury is not limited to partial or total sectioning but may also be attributable to the neuropraxia that results from compression, traction, coagulation, ischemia, and inflammation of the tissues in the NVB region [3], [4]. In addition, arterial insufficiency resulting from injury to the lateral and apical accessory pudendal arteries during RP may play a role in the pathophysiology of postprostatectomy ED [5].

To enhance the recovery of erectile function after RP, different strategies for penile rehabilitation have been investigated, including intracavernosal injection of prostaglandin E1, phosphodiesterase type 5 inhibitors (PDE5Is), intraurethral and topical alprostadil, and vacuum erectile devices. However, after 25 yr, the optimal penile rehabilitation strategy is still a matter of debate [6].

Preclinical studies showed that low-intensity extracorporeal shockwave therapy (LI-ESWT) enhances tissue regeneration via its shear stress effect (microtrauma and mechanical stress effects on deep tissue), which subsequently increases the expression of VEGF and endothelial nitric oxide synthase, causing tissue neoangiogenesis and thus improving its blood flow [7]. Subsequently, there was an increase in interest in the use of LI-ESWT in the management of vasculogenic ED [3]. In 2016, Li et al. [8] reported for the first time that LI-ESWT could potentially improve erectile function in Sprague-Dawley rats with bilateral cavernous nerve injury via activation of Schwann cell proliferation and an increase in neuronal nitric oxide synthase. Subsequently, several authors assessed the impact of this energy on the treatment of patients with postprostatectomy ED [3], [4], [9], [10], [11], [12], [13], [14], [15]. The aim of the current study was to systematically review the literature to assess the value of LI-ESWT in the management of patients with postprostatectomy ED.

## 2 Evidence acquisition

### 2.1 Search strategy

**Fig. 1:**
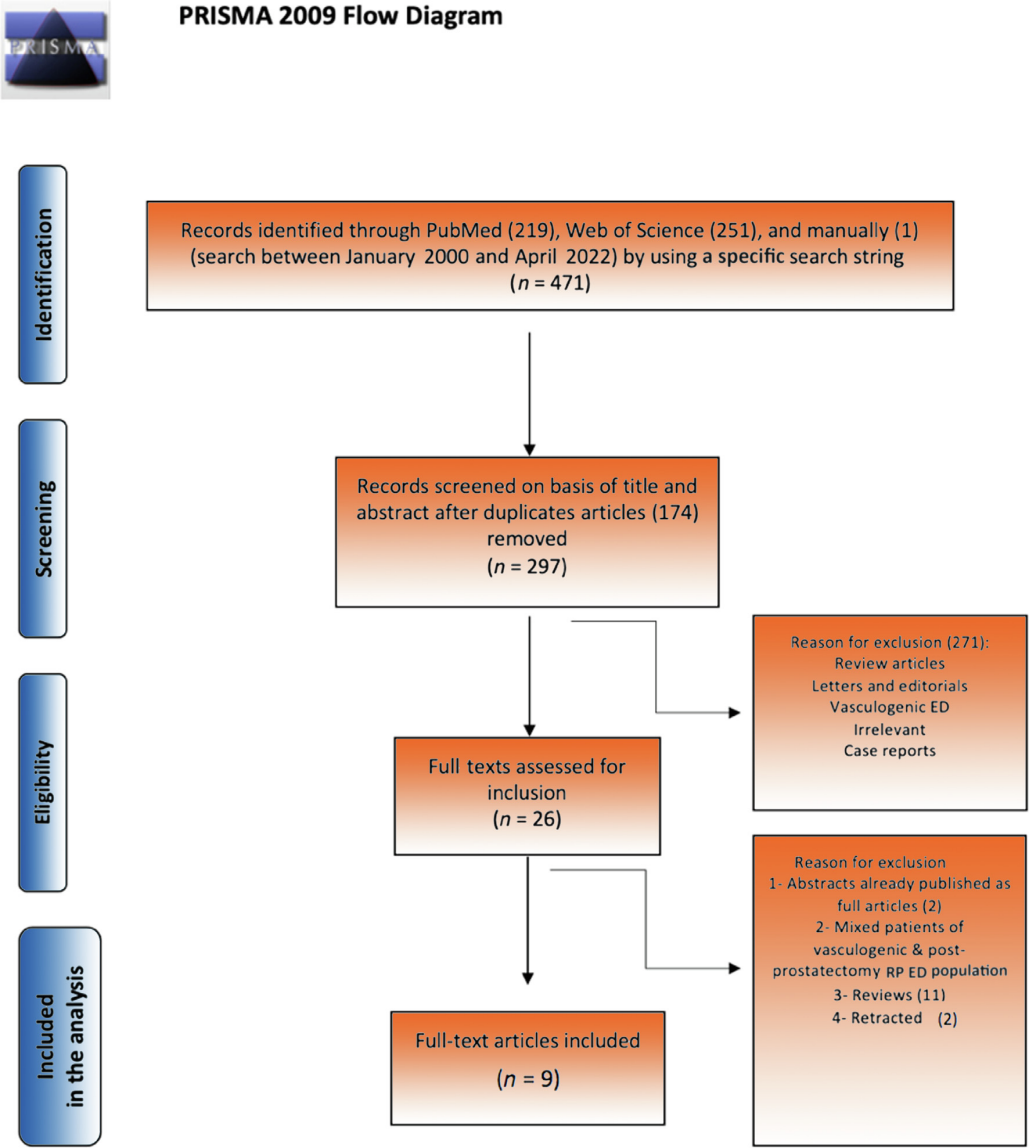
Flow diagram showing study inclusion and exclusion. PRISMA = Preferred Reporting Items for Systematic Reviews and Meta-Analyses; ED = erectile dysfunction; RP = radical prostatectomy.

A systematic search of the PubMed and Web of Science databases in April 2022 performed by two authors (A.E. and M.E.) using a combination of different keywords (Supplementary material) identified 471 reports, of which 174 were excluded as they were duplicates (Fig. 1). Screening of the remaining 297 articles by title and abstract resulted in exclusion of 271 (irrelevant, vasculogenic ED, reviews, letters, editorials, and case reports). Full-text assessment was performed for the remaining 26 articles. Finally, nine articles were included in the final review (Table 1). The review was carried out in accordance with the Preferred Reporting Items for Systematic Reviews and Meta-Analyses (PRISMA) statement (Supplementary material) [16].

**Table 1:** Summary of the studies included in the review

Study	*N*	Patients’ characteristics	Protocol	ESWT machine	Sessions (*n*)	Time/session	Regions	No. of waves	Frequency	EFD (mJ/mm^2^)	Outcomes	Notes
Jang 2022 [15]	39	Age: 66 yr PSA: 6.32 ng/ml IIEF-5 score: 18 BMI: 24.36 kg/m^2^ DM: 30.8% EHS: 3	Tadalafil 5 mg starting from week 1 to 6 mo after RP	NA	NA	NA	NA	NA	NA	NA	**EHS ≥3** 3 wk: 0% 1 mo: 5.1% 3 mo: 5.1% 6 mo: 10.3%	No significant difference between the groups except at 6 mo (*p* = 0.034) On multivariate logistic regression LI-ESWT was the only significant predictor of EHS ≥3 at 6 mo
	41	Age: 62 yr PSA: 7 ng/ml IIEF-5 score: 19 BMI: 24.88 kg/m^2^ DM: 14.6% EHS: 3	Tadalafil 5 mg starting week 1 to 6 mo after RP + LI-ESWT on days 4, 5, 6, and 7 and in weeks 2 and 4 after RP	Omnispec ED1000 (EH)	6	15 min	Right crus Left crus 1/3 root 1/3 middle 1/3 tip	1500 (300 per region)	120/min	0.09	**EHS ≥3** 3 wk: 14.6% 1 mo: 12.2% 3 mo: 14.6% 6 mo: 29.3%	
Porst 2021 [3]	12	NA	Tadalafil 5 mg, starting 5 d pre RP + LI-ESWT 8–14 d after RP	Dornier Aries 2 (EM) MTS Urogold 100 (EH) PiezoWave (PE)	6–10	NA	NA	NA	NA	Up to 0.30	Success rate 83.3%	10 patients returned to preRP IIEF; 2 patients reported failed treatment, but had impaired EF before RP
Karakose 2021 [10]	32	Age: 58.4 ± 6.7 yr PV: 34.5 ± 15.3 ml PSA: 9.9 ± 7.8 ng/ml IIEF-5 score: 21 ± 3.6 BMI: 28.7 ± 4.8 kg/m^2^ DM: 12.5% HTN: 21.8% CAD: 18.7%	Only tadalafil 5 mg starting on day 3 after RP	–	–	–	–	–	–	–	**IIEF-5 score** 3 mo: 7 ± 2.8 6 mo: 7 ± 2.9 12 mo: 9 ± 3.4	IIEF-5 was assessed at 3, 6, and 12 mo after RP.
	34	Age: 59.2 ± 6.8 yr PV: 35.1 ± 17.2 ml PSA: 9.9 ± 5.3 ng/ml IIEF-5 score: 21 ± 2.8 BMI: 28.4 ± 5.1 kg/m^2^ DM: 14.7% HTN: 23.5% CAD: 17.6%	Tadalafil 5 mg starting on day 3 after RP + LI-ESWT (2 sessions/wk starting 3 mo after RP)	Omnispec ED1000 (EH)	12	15 min	Right crus Left crus 1/3 root 1/3 middle 1/3 tip	1500 (300 per region)	160/min	0.09	**IIEF-5 score** 3 mo: 7 ± 2.2 6 mo: 13 ± 3.3 12 mo: 18 ± 3	
Inoue 2020 [12]	5	Age: 62.2 ± 2.68 PV: 25.2 ± 5.76 ml PSA: 4.95 ± 2.82 ng/ml SF score: 66.5 SB score: 86.3 BMI: 24.5 ± 1.25 kg/m^2^ T: 4.24 ± 0.78 ng/ml NSS: 20%	Early LI-ESWT of 3 sessions/wk for the first 2 wk after RP then once weekly for 6 wk	Omnispec ED1000 (EH)	12	20 min	Right crus Left crus 1/3 root 1/3 middle 1/3 tip	1500 (300 per region)	120/min	0.09	**SF score** 6 mo: 19.2 9 mo: 20.9 12 mo: 28 **SB score** 3 mo: 33.8 6 mo: 46.3 9 mo: 61.3 12 mo: 56.3	SF and SB were assessed using the EPIC score at 0, 3, 6, 9, and 12 mo after RP
	11	Age: 62.9 ± 1.80 yr PV: 25.2 ± 5.76 ml PSA: 6.39 ± 1.90 ng/ml SF score: 41.8 SB score: 69.9 BMI: 23.3 ± 0.84 kg/m^2^ T: 4.82 ± 0.58 ng/ml NSS: 63.6%	Delayed LI-ESWT starting 6 mo after RP: 2 sessions/wk for 3 wk, followed by 3 wk of rest, then 2 sessions/wk for 3 wk	Omnispec ED1000 (EH)	12	20 min	Right crus Left crus 1/3 root 1/3 middle 1/3 tip	1500 (300 per region)	120/min	0.09	**SF score** 6 mo: 17.9 9 mo: 25.8 12 mo: 21.3 **SB score** 3 mo: 41.9 6 mo: 54.2 9 mo: 71.9 12 mo: 82.3	
	178	Age: 66.6 ± 0.45 yr PV: 29.2 ± 0.97 ml PSA: 9.12 ± 0.47 ng/ml SF score: 31.8 SB score: 87.9 BMI: 23.4 ± 0.21 kg/m^2^ T: 4.66 ± 0.13 ng/ml NSS: 43.8%	No LI-ESWT	–	–	–	–	–	–	–	**SF score** 6 mo: 8.1 9 mo: 10.2 12 mo: 9.5 **SB score** 3 mo: 68.3 6 mo: 71.3 9 mo: 68.9 12 mo: 68.8	
Baccaglini 2020 [4]^a^	41	Age: 64.6 ± 5.3 yr BMI: 25.9 ± 2.7 kg/m^2^ HTN: 53.7% DM: 19.5% Smoking: 4.9% IIEF-5 score: 22	5 mg/d radalafil after removal of urethral catheter (7 –10 d)	–	–	–	–	–	–	–	**At 16 wk** IIEF-5: 10 IIEF-5 ≥17: 17.1%	The difference in IIEF-5 score was significant at the last follow-up visit, but did not reach the primary clinical endpoint of a difference of ≥4 points
	36	Age: 64.6 ± 5.3 yr BMI: 26.6 ± 3.6 kg/m^2^ HTN: 63.9% DM: 16.7% Smoking: 8.3% IIEF-5 score: 21	5 mg/d tadalafil after removal of urethral catheter (7–10 d) + LI-ESWT beginning 6 wk after RP	Renova (DIREX group) (EM)	8	8 min	Right crus Left crus 1/3 root 1/3 middle 1/3 tip	2400 (600 per region)	300/min	0.09	**At 16 wk** IIEF-5: 12 IIEF-5 ≥17: 22.2%	
Ladegaard 2020 [9]^a^	20	Age: 60.8 yr BNS: 35% UNS: 65% CAD: 55% DM: 10% Smokers: 15% PDE5I: 90% EHS score: 1.30 IIEF-5 score: 6.8	One LI-ESWT session/wk for 5 wk	Duolith SD1 (EM)	5	NA	Right crus Left crus 1/3 root 1/3 middle 1/3 tip	4000	300/min	0.15	**At 4 wk** EHS: +0.35 IIEF-5: +2.4 **At 12 wk** EHS: +0.5 IIEF-5: +3.45	NA
	18	Age: 64.3 yr BNS: 28% UNS: 72% CAD: 33% DM: 22% Smokers: 17% PDE5I: 89% EHS score: 1.44 IIEF-5 score: 6.83	Sham protocol	–	–	–	–	–	–	–	**At 4 wk** EHS: −0.17 IIEF-5: +1.28 **At 12 wk** EHS: −0.17 IIEF-5: +0.65	
Zewin 2018 [14]^a^	42	Age: 52.9 ± 7.2 yr BMI: 27.2 ± 1 kg/m^2^ Smokers: 21.4% IIEF score: 68.5	Two LI-ESWT sessions/wk for 3 wk, repeated after 3 wk of no treatment	Dornier Aries (EM)	12	15 min	Right crus Left crus 1/3 root 1/3 middle 1/3 tip	1500 (300/ region)	120/min	0.09	**IEEF score** 1 mo: 19.4 3 mo: 55.7 6 mo: 59.9 9 mo: 60.7	16% more patients in the LI-ESWT group and 19% in the PDE5I group reported potency recovery compared to the sham group; this finding was not statistically significant but it was of clinical importance
	43	Age: 53.4 ± 5.9 yr BMI: 25.3 ± 0.9 kg/m^2^ Smokers: 14% IIEF score: 68.8	PDE5I (sildenafil 50 mg/d for 6 mo)	–	–	–	–	–	–	–	**IEEF score** 1 mo: 19.3 3 mo: 55.9 6 mo: 60.7 9 mo: 61.5	
	43	Age: 51.2 ± 6.3 yr BMI: 26.8 ± 1.2 kg/m^2^ Smokers: 16.3% IIEF score: 68.6	No treatment	–	–	–	–	–	–	–	**IEEF score** 1 mo: 19.5 3 mo: 53.7 6 mo: 56.4 9 mo: 56.7	
Frey 2015 [13]	6	Age: 62 yr **IIEF-5 score** 25 before surgery 9.5 before ESWT	Two sessions per week every other week for 6 wk	DuoLith SD1 T-Top (EM)	6	NA	Root Shaft Glans	3000	300/min	0.20 0.15 0.12	**IIEF-5 score** 1 mo: +3.5 12 mo: +1	At 12 mo, 3 patients discontinued their erectogenic aids
Ericson 2020 [11]^b^	29	Age: 62.5 yr **Pre-ESWT score** **(6 wk post-RP)** SHIM: 5 EHS: 1	PDE5I only	–	–	–	–	–	–	–	**12 wk** SHIM: 6 EHS: 1 **24 wk** SHIM: 5 EHS: 1	Normal EF reported by 25% of patients at 3 mo
	23	Age: 59.2 yr **Pre-ESWT score** **(6 wk post-RP)** SHIM: 7 EHS: 2	Once weekly sessions over a period of 6 wk started 2 wk after RP + PDE5I	Zimmer enPuls 2.0 (EM)	6	NA	Corpora and cavernosal bundle bilaterally	10,000	NA	0.09	**12 wk** SHIM: 7 EHS: 2 **24 wk** SHIM: 10 EHS: 2	Normal EF reported by 36.4% of patients at 3 mo

BMI = body mass index; BNS = bilateral nerve-sparing; CAD = coronary artery disease; DM = diabetes mellitus; EF = erectile function; EFD = energy flux density; EH = electrohydraulic; EHS = Erection Hardness Score; EM = electromagnetic; EPIC = Expanded Prostate Cancer Index Composite; HTN = hypertension; IIEF = International Index of Erectile Function; LI-ESWT = low-intensity extracorporeal shockwave therapy; NA = not applicable; NSS = nerve-sparing surgery; PDE5I = phosphodiesterase type 5 inhibitor; PE = piezoelectric; PSA = prostate-specific antigen; PV = prostate volume; RP = radical prostatectomy; SB = sexual bother score from EPIC; SF = sexual function score from EPIC; SHIM = Sexual Health Inventory for Men; T = testosterone; UNS = unilateral nerve-sparing.

a Randomized controlled trial.

b Conference abstract.

### 2.2 Quality assessment

Assessment of the risk of bias is reported and discussed in the Supplementary material.

## 3 Evidence synthesis

### 3.1 Characteristics of the studies included

All the studies included were published between 2015 and 2022. Only three studies were randomized controlled trials (RCTs), of which two compared the combination of PDE5Is and LI-ESWT versus PDE5Is alone [4], [14] and one compared LI-ESWT versus a sham protocol in the management of postprostatectomy ED [9]. Furthermore, three reports were nonrandomized comparative studies [10], [12], [15] and two reports described noncomparative experience with LI-ESWT use in single centers for postprostatectomy ED [3], [13]. One conference abstract was included owing to the scarcity of data on this topic in the literature [11].

In terms of the geographic distribution of the studies, two were carried out in Denmark [9], [13] and one in each of Egypt [14], Brazil [4], Turkey [10], Germany [3], South Korea [15], Japan [12], and the USA [11]. The authors reported no conflicts of interest and an absence of funding, except for the study by Frey et al. [13], for which the authors declared their potential conflicts of interest and that the ESWT machine was provided by Storz Medical.

### 3.2 LI-ESWT for postprostatectomy ED

In 2013, Inoue et al. [17] reported on LI-ESWT use for the management of five middle-aged men (mean 63.2 yr) with ED, of whom three had ED following laparoscopic RP and two suffered from vasculogenic ED. The authors reported that LI-ESWT has the potential to significantly improve the Erectile Hardness Score (EHS) for patients suffering from vasculogenic ED but not for patients with ED following laparoscopic RP. However, it should be noted that the three RP patients in this study underwent non–nerve-sparing RP [17]. These findings were confirmed by Chung et al. [18], who included 3/30 patients suffering from ED after RP and reported that LI-ESWT was associated with a statistically significant improvement in erectile function for patients with vasculogenic ED in comparison to those with postprostatectomy ED. In their pioneering work in 2015, Frey et al. [13] demonstrated that LI-ESWT can potentially improve erectile function in patients with ED following bilateral nerve-sparing RP, which raised urologists’ interest in investigating the value of LI-ESWT in patients with postprostatectomy ED [2], [3], [4], [9], [10], [11], [12], [13], [14], [15].

There is no standardized LI-ESWT protocol for patients with postprostatectomy ED. For instance, some authors reported a protocol consisting of one session weekly [4], [9], [11], while others reported two sessions [10], [12], [13], [14] or even up to three sessions per week [12], [15]. Similarly, the total number of sessions (ranging from 5 sessions [9] to 12 [10], [12], [14]), the number of shockwave per session (ranging from 1500 [10], [12], [14], [15] to 10 000 [11]), and the wave frequency (ranging from 120 [12], [14], [15] to 300 [4], [9], [13] waves/min) were highly variable. Most studies divided the number of shockwaves administered per sessions over five main regions of the penis, consisting of the right crura, the left crura, the root, the shaft, and the tip of the penis [4], [10], [12], [14], [15], except for Ladegaard et al. [9], who split the shockwaves over eight regions by dividing the penile root, shaft, and tip into right and left regions. Energy flux density (EFD), defined as energy per area, of 0.09 mJ/mm^2^ was the setting most commonly used [4], [10], [11], [12], [14], [15], while higher EFD settings (0.15–0.30 ml/mm^2^) were reported in three studies [3], [9], [13]. These protocol heterogeneities can be explained by the use of different shockwave machines with different energy sources. Porst [3] reviewed shockwave machines used for the treatment of ED and found that each device provides a different EFD range that yields different total energy per shot and thus a different experience for the patient.

All the studies included in our review evaluated middle-aged men (mean age ranging from 52.9 yr [14] to 66.6 yr [12]) suffering from postprostatectomy ED mainly secondary to nerve-sparing RP. Only one RCT assessed the value of LI-ESWT in the management of ED secondary to nerve-sparing radical cystoprostatectomy [14]. The authors randomized patients to receive LI-ESWT, PDE5Is, or no treatment at all. Although they found no statistically significant differences among the three groups, LI-ESWT and PDE5Is were associated with 16% and 19% better recovery of potency, respectively, when compared to the no-treatment group [14].

Several authors compared a penile rehabilitation protocol consisting of a combination of LI-ESWT and PDE5Is versus PDE5Is alone, reporting a significant improvement in erectile function with the combination protocol when compared to the PDE5Is protocol [3], [4], [9], [10], [11], [15]. This finding was further confirmed when considering only the results from RCTs [4], [9]; however, it should be noted that not all the statistically significant findings are of clinical importance, as reported by Baccaglini et al. [4], who demonstrated that the proportion of patients achieving a clinically significant International Index of Erectile Function-5 (IIEF-5) score (>17) was not statistically significant at 4 mo (control group 17.1% vs experimental group 22.2%; *p* = 0.57). This finding is in line with the outcomes reported by Ericson et al. [11], who demonstrated similar results (36.4% for PDE5Is + LI-ESWT vs 25% for PDE5Is alone; *p* = 0.51). Similarly, Jang et al. [15] reported that only 29.3% of patients experienced a clinically significant improvement in EHS (≥3) at 6 mo in a cohort undergoing penile rehabilitation with a LI-ESWT + PDE5Is protocol, compared to 10.3% of patients using only PDE5Is. However, these studies had small sample sizes and short follow-up.

The most recent meta-analysis of RCTs on penile rehabilitation protocols after radical prostatectomy reported that only regular sildenafil 100 mg intake (nightly or daily doses) and pelvic floor muscle training were associated with enhanced recovery of potency, yet none of the studies included in our review reported on the use of sildenafil 100 mg or pelvic floor muscle training in their penile rehabilitation protocol [6]. Most authors reported daily intake of tadalafil 5 mg [3], [4], [10], [15], except one study that used sildenafil 50 mg daily [14].

The starting time for LI-ESWT differed among the studies, ranging from 3 d [10] to 6 mo [12] after surgery. Inoue et al. [12] suggested that early LI-ESWT application may improve neural recovery by enhancing cavernosal blood flow and preventing penile remodeling, while late LI-ESWT may play an important role in reversing penile fibrotic remodeling [12]. The authors compared early (starting 1–2 wk after surgery) versus delayed (starting 6 mo after surgery) LI-ESWT and found that early LI-ESWT sessions before catheter removal were associated with better recovery of sexual function when compared to the delayed protocol. However, this study has many limitations, including a small sample size and an inability to control confounding factors such as the percentage of patients undergoing nerve-sparing surgery (20% in the early protocol group vs 63.3% in the delayed protocol group) [12].

To date, there is no standardized tool for reporting sexual function outcomes following RP, which represents an obstacle towards defining the ideal management option for postprostatectomy ED. It is noteworthy that not all statistically significant improvements in results for the tools currently available truly reflect patients’ satisfaction and perception of treatment success [19]. The studies included showed high heterogeneity regarding the tools used for assessment of postoperative erectile function. Some studies used only IIEF-5 [4], [10], [13], EHS alone [15], a combination of EHS and IIEF-5 [9], [11], or other measures (such as sexual bother and sexual function scores assessed as a part of the Expanded Prostate Cancer Index Composite questionnaire and IIEF) [12], [14]. Regardless of the tool used, return of sexual function to the preoperative baseline is considered the most realistic measure of sexual function following RP [19].

Incontinence is another distressing problem that may occur after RP. Only two studies evaluated the hypothesis that LI-ESWT may affect postoperative continence function, and reported no significant difference between patients receiving LI-ESWT and those who did not [4], [10]. Similarly, Zewin et al. [14] reported no significant difference in continence function following radical cystoprostatectomy among LI-ESWT, PDE5Is, and control groups.

The current review is not devoid of limitations. First, owing to the scarcity of studies, we decided to include a conference abstract that did not provide all the data required for full evaluation of the study. Second, most of the observational studies included in the review were characterized by high risk of bias and short follow-up. Third, the sample size in almost all of the studies included is small, with a cumulative total of 230 patients across all the studies published in the literature. Fourth, we did not perform a meta-analysis because of the scarcity of studies addressing this topic and the high heterogeneity among the studies included in the review. Furthermore, it was not possible to discuss the clinical outcomes of LI-ESWT in relation to the treatment protocol owing to the high heterogeneity in terms of the tools used for assessment of erectile function and patient follow-up. Finally, not all of the studies included used LI-ESWT in a penile rehabilitation setting, as some studies used LI-ESWT for the treatment of postprostatectomy ED. However, to the best of our knowledge, this is the first systematic review in the literature addressing this topic.

## 4 Conclusions

There is currently limited evidence in the literature on the use of LI-ESWT either alone or in combination with PDE5Is in penile rehabilitation protocols after RP. However, initial results obtained from preclinical studies on animal models and small clinical trials with short follow-up show that LI-ESWT may potentially play a role in the management of postprostatectomy ED in the future. Further RCTs with large sample sizes are required to support these findings.

  ***Author contributions***: Maria Chiara Sighinolfi had full access to all the data in the study and takes responsibility for the integrity of the data and the accuracy of the data analysis.

*Study concept and design*: Sighinolfi, Eissa, Bellorofonte, Mofferdin, Eldeeb.

*Acquisition of data*: Sighinolfi, Eissa, Bellorofonte, Mofferdin, Eldeeb, Assumma, Panio, Calcagnile.

*Analysis and interpretation of data*: Sighinolfi, Eissa, Eldeeb, Assumma, Panio, Calcagnile, Stroppa.

*Drafting of the manuscript*: Sighinolfi, Eissa.

*Critical revision of the manuscript for important intellectual content*: Bozzini, Gaia, Terzoni, Sangalli, Micali, Rocco.

*Statistical analysis*: Eissa, Eldeeb.

*Obtaining funding*: None.

*Administrative, technical, or material support*: None.

*Supervision*: Bozzini, Gaia, Micali, Rocco.

*Other*: None.

  ***Financial disclosures:*** Maria Chiara Sighinolfi certifies that all conflicts of interest, including specific financial interests and relationships and affiliations relevant to the subject matter or materials discussed in the manuscript (eg, employment/affiliation, grants or funding, consultancies, honoraria, stock ownership or options, expert testimony, royalties, or patents filed, received, or pending), are the following: None.

  ***Funding/Support and role of the sponsor:*** None.
