# Supplementary material for: Low-intensity Extracorporeal Shockwave Therapy for the Management of Postprostatectomy Erectile Dysfunction: A Systematic Review of the Literature
Source: Eur Urol Open Sci. 2022 Jul 30;43:45–53. doi: 10.1016/j.euros.2022.07.003 (PMC9344341; doi:10.1016/j.euros.2022.07.003)
Supplement: Supplementary Data 1 [file mmc1.docx]

**Supplementary material**

**Search process**

The current review was carried out in accordance with the Preferred Reporting Items for Systematic Reviews and Meta-Analyses (PRISMA) statement. Two authors (AE and ME) independently performed a systematic search of the PubMed and Web of Science databases in April 2022 using the below listed keywords to identify all the relevant articles. Any disagreement among the two authors were resolved (by MCS). Initially articles were screened through the evaluation of the title and the abstract to exclude irrelevant studies, subsequently, full text assessment of the remaining articles was performed. Furthermore, the references of all the included reports were searched manually to include any missed studies.

*Inclusion and exclusion criteria*

There was no restriction on the type of study (retrospective or prospective), the minimum number of patients, ED severity, the presence of control arm, the tool used for assessment of erectile function, and the use of concomitant erectogenic pharmaceutical. Furthermore, conference abstracts were included in the present review provided that the final version of the study was not yet published, and the authors provide sufficient information about their methodology and results. Finally, non-English reports, case reports, reviews, letters, and editorials were excluded.

*Data Extraction*

Two authors (AE and ME) extracted the following data from the included articles: year of publication, type of the study, funding, patients characteristics (age, body mass index, comorbidities, and baseline erectile function), ED treatment protocol, ESWT machine, type of waves (focused versus linear), number of sessions, time of each session, number and frequency of shockwaves, target regions of the shockwaves, and the outcomes. All data were collected in an excel sheet (Microsoft Corporation, Redmond, CA, USA). After data extraction, quantitative analysis was not possible due to the very high heterogeneity among the included studies and only qualitative analysis was carried out.

**Search strings**

1- Pubmed “Filters: English”

**Keywords**

("LiESWT" OR "ESWT" OR "extracorporeal shockwave therapy" OR "extracorporeal shock wave therapy" OR "extracorporeal shock-wave therapy" OR "penile rehabilitation") AND ("prostatectomy" OR "cystectomy" OR "cystoprostatectomy" OR "prostate cancer")

2- Web of Science “Filters: English, articles, early access”

**Keywords**

TS=(LiESWT OR ESWT OR extracorporeal shockwave therapy OR extracorporeal shock wave therapy OR extracorporeal shock-wave therapy OR penile Rehabilitation) AND TS=(prostatectomy OR cystectomy OR cystoprostatectomy OR prostate cancer)

**Risk of bias assessment**

Risk of bias assessment was performed independently by two authors (AE and MCS) following the GRADE guidelines for observational studies and RCTs [1]. Generally, the GRADE guidelines for observational studies are based on the assessment of four main types of bias including the bias in eligibility criteria, measurement of exposure and outcomes, controlling confounding, and follow up bias. Considering RCTs, the guidelines assess the presence of any of the following limitations: lack of allocation concealment, blinding, incomplete accounting of patients and outcomes, reporting bias, and other limitations [1].

The risk of bias assessment for the observational studies is presented in Supplementary Figure 1. Generally, most of the included observational studies show a high risk of bias with the most common limitation being the short follow up period (maximum 12 months). As regards the RCTs, the risk of bias assessment was lower compared to the observational studies; however, they share the same limitation of the short follow up period (maximum 9 months). Furthermore, none of the included RCTs commented on the allocation concealment (Supplementary Figure 2).

**References**

[1] Guyatt GH, Oxman AD, Vist G, Kunz R, Brozek J, Alonso-Coello P, et al. GRADE guidelines: 4. Rating the quality of evidence—study limitations (risk of bias). J Clin Epidemiol 2011;64:407–15. https://doi.org/10.1016/j.jclinepi.2010.07.017.

**Supplementary Figure 1:** Risk of bias and study limitations according to GRADE guidelines for observational studies

**Supplementary Figure 2:** Risk of bias and study limitations according to GRADE guidelines for randomized controlled trial
